# Supplementary material for: Improving quality of preclinical academic research through auditing: A feasibility study
Source: PLoS One. 2020 Oct 15;15(10):e0240719. doi: 10.1371/journal.pone.0240719 (PMC7561085; doi:10.1371/journal.pone.0240719)
Supplement: S1 File — (PDF) [file pone.0240719.s001.pdf]

Audit Plan – Internal / External Audit

Type of Audit	
Organization	
Site to be audited	
Scope	
Date	
Contact Person	
Auditors	

Time Schedule	Content	Auditors / Participants
From:<br>To:	**Welcome and Opening Dialog**<br>Define Procedure of the Audit	
From:<br>To:	**Content of the Audit**<br><br>Objectives:<br>______________________________________<br>______________________________________<br>______________________________________<br>______________________________________<br>______________________________________<br><br>Subjects:<br>□ Procedures (SOPs / Hands-on): Which ones?<br>□ Resources, roles & responsibilities<br>□ Primary data management<br>□ Projects (Which one<br>□ Experimental plan<br>□ Analysis plan & results<br>□ Programs (Documentation)	
From:<br>To:	**Auditor(s) evaluation of the results** (Meeting Auditors) for preliminary communication to the audited site.	
From:<br>To:	**Final post-audit discussion of the results** with the audited site.	
Not later than one week after the audit	**Draft of the audit results** sent to the audited site for review	
Not later than one week after sending the draft	**Final report** agreed and available to all parties	
