# Supplementary material for: Improving quality of preclinical academic research through auditing: A feasibility study
Source: PLoS One. 2020 Oct 15;15(10):e0240719. doi: 10.1371/journal.pone.0240719 (PMC7561085; doi:10.1371/journal.pone.0240719)
Supplement: S2 File — (PDF) [file pone.0240719.s002.pdf]

Sample Template	Checklist Internal and External Audit

This checklist is an example of a possible implementation of internal and external audits in the field of basic biomedical research in experimental neurology. It has been designed as a practical tool for conducting different types of audits, but it must be adapted to the context of the audited organization. The checklist makes no claim to be complete.

Organization:
Date:
Participants in the Audit:
Auditor(s):

Table A: Internal Audits

**Method Audit**	**Questions**
Aim of the Audit	What is expected to be achieved?
Method	Which is the experimental method to be audited? What is the objective or read out of the method?
Application	Who applies the method? Are the people applying the method appropriately trained?
Infrastructure / Resources	Is the working environment suitable for the method to be applied? Are all necessary working materials and reagents available? Are the instruments and measuring equipment calibrated and maintained? Are the chemicals and other consumables used within their shelf life?
Risk	Are there any risks in the implementation of the method? Are strategies in place to mitigate them?
Method Comparison	Ask several people performing the method to describe the steps of the method. Is a protocol available? If so, how did it come about? At what point are differences noticeable between operators?
Documentation	How and where are the data and results documented and analyzed? Is the documentation traceable?
Changes	Does the SOP (protocol) have to be changed? If so, at what points and why?
Improvement	How could the method be improved?
Corrective Activities	Are the corrective actions taken for the method verified for their effectiveness?
Key Performance Indicators KPI	Is there a performance indicator for the method? If so, what is the benefit of the key figure? Can a statement be made with the KPI?
Knowledge Sharing	How is the method passed on and further developed?
Trainings	Is the implementation of the method trained? Who is responsible? In which periods do trainings take place?
**Data Audit**	**Questions**
Aim of the Audit	What is expected to be achieved?
Data	What data will be audited? Are the data primary or secondary data?
Generation	How was the data collected? Is there a protocol or standards operating procedure (SOP) for this?
Infrastructure / Resources	Is the working environment in which the data was generated appropriate? Are all necessary working tools and software available? Is the software validated? Are the licenses currently valid?
Risk	Are there any risks in the generation of the data? Are strategies in place to mitigate them?
Storage	How and where is the data stored? Is the storage path within the working group consistent and known to everyone? Do all employees adhere to the specifications?

Sample Template	Checklist Internal and External Audit
Documentation	How and where is the data documented, analyzed and evaluated? What methods are used?
Changes	Did the storage of the data have to be changed? If so, for what reason? Where is it stored now?
Improvements	How could an improvement in data collection and/or storage look like?
Key Performance Indicators KPI	Is there a performance indicator for data collection? If so, what is the benefit of the key figure? Can a statement be made with the KPI?
Open Data / Open Access	Will the data be published as open data and open access? If not, why not? Is the implementation known to all parties involved?
**Document Audit**	**Questions**
Aim of the Audit	What is expected to be achieved?
Documents	What documents are generated in the area? What are the requirements? Is a Good Documentation Practice available? Are its requirements fulfilled? How does the change process for the documents look like?
Trainings	Are the staff trained in Good Documentation Practice?
Archiving	What documents are archived? Where and how long? Are there any legal requirements for this?
Uses	Who are the users of the documents? Is the usage known to everyone?
Infrastructure / Resources	Who has “read” and “write” access? How are the documents marked, versioned and updated? Is there a document control system available? Which one?
Risk	Are there risks in document control? Are they avoided?
Changes	Has the documentation practice or the document control been changed? If so, at which points and why?
Improvements	How could the documentation be improved?
Key Performance Indicators KPI	Is there a performance indicator for documentation? If so, what is the benefit of the key figure? Can a statement be made with the KPI?
Knowledge Sharing	How is the documentation passed on?
**Process Audit**	**Questions**
Aim of the Audit	What is expected to be achieved?
Process	Which is the process is to be audited? What is the goal of the process? What are the requirements for the process?
Users	Who are the users of the process? Is its application known to everyone involved?
Responsibilities	How are the responsibilities within the process defined? Are they followed? Are there gaps? If so, why?
Infrastructure / Resources	Is the working environment in which the process is to be performed appropriate? Are all necessary working materials available? Are the devices and measuring equipment calibrated and maintained?
Risk	Are there any risks in the process? Are strategies in place to mitigate them?
Description	Is a suitable description for the process? If not, why not? If so, what is the situation? Have plausibility checks been carried out?
Documentation	How and where is the process documented? Is it traceable? Is there a protocol for the process? Is it standardized?
Requirements	Are the requirements placed on the process fulfilled? If not, why not?
Changes	Does the process need to be changed? If so, at what points and why?
Improvements	How could the process be improved?
Key Performance Indicators KPI	Is there a performance indicator for the process? If so, what is the benefit of the key figure? Can a statement be made with the the KPI?
Knowledge sharing	How is the knowledge about the implementation of processes / passed on and further developed?

Table B: External Audit

Peer Audit	Questions
Aim of the Audit	What is expected to be achieved?
People involved	Who are the persons / groups involved in the Peer Audit?
Review of Projects and Processes	Which project or process is being reviewed? Are methods be compared? If so, which ones?
Uses	Who is involved in the audited processes or projects? Is their participation known to the project responsible?
Responsibilities	How are the responsibilities within the project/process defined? Are they observed? Are there gaps? If so, why?
Infrastructure / Resources	Is the working environment in which the process take place appropriate? Are all necessary working materials available? Are the relevant devices and measuring equipment calibrated and maintained?
Risks	Are risks known which have influenced the project/process and which could have been avoided in advance?
Project (Study) Planning	Was a complete project planning carried out before starting the experiments? Is a suitable study design available for the project/process? If not, why not? If so, what is the situation? Has a suitable hypothesis / counter-hypothesis been formulated? Has a plausibility check been carried out? Is the entire project planning traceable and documented?
Pre-registration	Is the project pre-registered? If not, why not?
Documentation	How is the project documented? Is the documentation comprehensible? Do SOPs or protocols exist for the implementation of the project?
Publication	Has the project already been published? Are the results reproducible? Are the evaluations and analyses comprehensible?
Changes	Should any changes in project planning, implementation, evaluation or reporting be done? If so, why?
Improvements	How could an improvement of the project look like?
Scientific Exchange	Experiences of the auditors on methods, implementation, evaluations etc. Were there any unusual features in the project? Discussion on plausibility and open questions that arose during the audit.
View from the Outside	Summary of the audit: what was positive, what should be improved? Hints and tips from the external colleagues. Possible emerging cooperation between auditors and audited.
