# Supplementary material for: Improving quality of preclinical academic research through auditing: A feasibility study
Source: PLoS One. 2020 Oct 15;15(10):e0240719. doi: 10.1371/journal.pone.0240719 (PMC7561085; doi:10.1371/journal.pone.0240719)
Supplement: S3 File — (DOCX) [file pone.0240719.s003.docx]

**S3 File**

All audits took place in the Department of Experimental Neurology of the Charité - Universitätsmedizin Berlin; the audits were not recruited by participants but by subjects.

For all assessments / audits the Department of Experimental Neurology, its applied methods were the subject of the audit.

The Internal Ring Audits, Peer Audit, Certification- and Monitoring Audits (ISO 9001) were led by personnel outside the Experimental Neurology Department.

Participation in audits and assessments was voluntary except for the external certification audit according to ISO 9001. The management of the department made them mandatory for all staff.

This study is a feasibility study and cannot be generalized to other research areas.

**S3_File_Table 5: Baseline characteristics of participants**

| **Assessments / Audits** | | **Method / Subject** | **Number / of performed Audits** | **Cumulative Number of audited Participants** | **Gender of audited Participants** | **Profession of Participants** | **Number of Auditors; Auditor’s qualification /profession** |
| --- | --- | --- | --- | --- | --- | --- | --- |
| **A**  **S**  **S**  **E**  **S**  **S M**  **E N T**  **S** | **Risk Assessment** | FMEA (1) | 3 | 3 | 2 males,  1 female | 3 scientists | 0 (self-assessment) |
|  | **Assessment of Failures and Errors** | LabCIRS (2) | 103 reported errors in six years | unknown; the system is based on anonymous reporting | male, female | technical assistants, students, scientists | 0 (self-assessment) |
| **I N**  **T**  **E**  **R N**  **A**  **L**  **A U**  **D**  **I**  **T**  **S** | **Audits of Methods, Data, Documentation, Processes** | Method Audit:  MCAO (3) | 2 | 6 | 3 males,  3 females | 6 scientists | 1; scientist |
|  |  | Method Audit:  in vivo: microcoil model mouse (4) | 1 | 4 | 2 males,  2 females | 1 technical assistant, 3 scientists | 1; scientist |
|  |  | Method Audit:  preparation of primary culture of rodent neurons | 2 | 6 | 1 male,  5 females | 2 technical assistant, 4 scientists | 2; scientists |
|  |  | Data Audit:  primary data storage | 10 | 1 / research group | 6 males,  4 females | 10 scientists | 2; scientists |
|  |  | Documentation Audits:  documentation of trainings, responsibilities, equipment, chemicals, samples | 5 | 6 | 2 males,  4 females | 2 technical assistant, 4 scientists | 1 per audit; QM trained scientist |
|  |  | Process Audit:  Sterile work conditions in in- vitro laboratory | 1 | 12 | 3 male, 9 females | 4 technical assistant, 1 student, 7 scientists | 2; scientists |
|  |  | Process Audit:  experimental /project-design Interleukin Quantification | 1 | 7 | 4 males,  3 females | 1 technical assistant, 6 scientists | 1; scientist |
|  | **Internal Ring Audits** | Ring Audits of the Charité | 3 | all staff (around 100 people) | male, female | technical assistants, students, scientists | 2 per audit; internal-audit-trained Charité employees |
| **E**  **X**  **T**  **E**  **R N**  **A**  **L**  **A U**  **D**  **I**  **T**  **S** | **Peer Audit** | experimental project design: Automatic Cell Quantification | 1 | 6 | 2 males,  4 females | 2 technical assistant, 4 scientists | 6; scientists |
|  | **Certification- and Monitoring Audits (ISO 9001)** | 1 certification audit,  2 monitoring audits | 3 | all staff (around 100 people) | male, female | technical assistants, students, scientists | 2 per audit; ISO certified external auditors |

1. FMEA = Failure mode and event analysis
2. LabCIRS = Laboratory Critical Incident Reporting System
3. MCAO = Middle cerebral artery occlusion in mice
4. Microcoil model mouse = Bilateral Common Carotid Artery Stenosis
