# Supplementary material for: Improving quality of preclinical academic research through auditing: A feasibility study
Source: PLoS One. 2020 Oct 15;15(10):e0240719. doi: 10.1371/journal.pone.0240719 (PMC7561085; doi:10.1371/journal.pone.0240719)
Supplement: Attachment — Submitted filename: Response to Reviewers3.docx [file pone.0240719.s004.docx]

Charité, Universitätsmedizin Berlin

Abteilung für Experimentelle Neurologie /

Quest Center of Berlin Institute of Health (BIH)

Charitéplatz 1

10117 Berlin	07.09.2020

**Answer to the editors**

Dear Editors,

The authors of the paper would like to thank you for your suggestion. We have responded to the question raised and have highlighted the changes in colour in this reply letter below.

If you have any further queries, please do not hesitate to contact us at any time.

Sincerely

Prof. Dr. Ulrich Dirnagl

In behalf of the authors.

**Issue**

1. Please ensure that you refer to Figure 2 in your text as, if accepted, production will need this reference to link the reader to the figure.

A graphical representation of the number of advantages and disadvantages of audits and assessments see **Figure 2.**

See Results p.13.

Revised.
